# Cancer-Alterome: a literature-mined resource for regulatory events caused by genetic alterations in cancer

**DOI:** 10.1038/s41597-024-03083-9

**Published:** 2024-03-02

**Authors:** Xinzhi Yao, Zhihan He, Yawen Liu, Yuxing Wang, Sizhuo Ouyang, Jingbo Xia

**Affiliations:** 1 Hubei Key Lab of Agricultural Bioinformatics, College of Informatics, Huazhong Agricultural University, Wuhan, 430070 P.R. China; 2 School of Computer Science and Engineering, Sun Yat-sen University, Guangzhou, 510006 P.R. China

**Keywords:** Tumour biomarkers, Data mining

## Abstract

It is vital to investigate the complex mechanisms underlying tumors to better understand cancer and develop effective treatments. Metabolic abnormalities and clinical phenotypes can serve as essential biomarkers for diagnosing this challenging disease. Additionally, genetic alterations provide profound insights into the fundamental aspects of cancer. This study introduces Cancer-Alterome, a literature-mined dataset that focuses on the regulatory events of an organism’s biological processes or clinical phenotypes caused by genetic alterations. By proposing and leveraging a text-mining pipeline, we identify 16,681 thousand of regulatory events records encompassing 21K genes, 157K genetic alterations and 154K downstream bio-concepts, extracted from 4,354K pan-cancer literature. The resulting dataset empowers a multifaceted investigation of cancer pathology, enabling the meticulous tracking of relevant literature support. Its potential applications extend to evidence-based medicine and precision medicine, yielding valuable insights for further advancements in cancer research.

## Background & Summary

Cancer, known as the primary cause of global mortality, has garnered significant attention in the field of pathological research. However, the interpretation and understanding of cancer pathology is an open challenge due to the complexity of the mechanism and diversity among individuals. The pathology of cancer is complex and it is characterized by intricate and dynamic interactions among molecular mechanisms. Furthermore, the pathology of cancer presents diversity among individuals, presenting multifaceted clinical features^1^.

The development of the Hallmarks of Cancer framework^2–4^ provided the possibility to alleviate the above challenges. Under this framework, the complex phenotypic and genotypic diversity of cancer has been distilled into a series of hallmark characteristics. These hallmarks, e.g., sustaining proliferative signaling, evading growth suppressors, and resisting cell death, were widely used to explain the mechanisms of tumor progression and therapy response. Initially proposed in 2000, this framework has undergone updates in 2011 and 2022. Through ongoing revisions, a multitude of diverse biological processes were incorporated into the cancer hallmark framework, indicating a long-term challenge of characterizing the molecular mechanisms underlying cancer pathology. Numerous studies investigated the underlying causes leading to the acquisition of these hallmarks, and the subsequent effect of the hallmarks. Skoulidis^5^ highlighted co-occurring genetic alterations that behaved as core determinants of the molecular and clinical heterogeneity of oncogene-driven lung cancer subgroups through their effects on both tumor cell-intrinsic and non-cell-autonomous cancer hallmarks. Bruggeman^6^ incorporated the expression of massive expression of germline cell-specific genes as cancer hallmarks, which actively promote tumor viability, proliferation, and metastasis. In addition, Kiefer^7^ mapped ten hallmarks of cancer to GO concepts and further associated them with upstream genes. By clustering individual hallmarks, they investigated the specific and overlapping hallmarks across diverse cancer types. In recent years, literature-based methods have been widely used to extract biological regulator events, aiming to extend the cancer hallmarks on a large scale. BioContext^8^, DigSee^9^ and GePI^10^ are known resources in this regard. Under this trend, a multitude of downstream biological processes and clinical features were discovered to facilitate the representation of molecular mechanisms of cancer.

Furthermore, the emergence of precision oncology in recent years has introduced an alternative approach to understanding and interpreting cancer pathology. Precision oncology is devoted to analyzing the biomarkers differences among individuals, and providing valuable insights into personalized diagnosis and treatment^11^. Current efforts in precision oncology have predominantly focused on identifying specific biomarkers such as genes and genetic alterations^1^. Established databases, such as the Catalogue of Somatic Mutations in Cancer (COSMIC)^12^, the Clinical Interpretation of Variants in Cancer (CIViC) database^13^, and the OncoKB precision oncology database^14^, were committed to providing comprehensive germline and somatic mutation information within the cancer progression. These databases also facilitate the assessment and interpretation of the clinical significance of these genetic alterations, thereby offering invaluable resources for personalized precision oncology. Furthermore, benefiting from advancements of text mining tools for biomedical entity recognition, a series of databases were developed to automate the curation of molecular mechanisms from massive literature data^15,16^. CancerMine^17^ associated genes and cancer pathology by identifying the roles of genes in the cancer progression, and collected precise sentence evidence for oncogenes, tumor suppressor genes, and driver genes. CIViCmine^18^, on the other hand, employed the text mining methods to extract corresponding literature support for gene mutations that were reported in the CIViC database, and enhanced the interpretability of cancer genetic alterations. Not only focusing on the standard single nucleotide polymorphisms (SNPs), CIViCmine identified and extracted general mentions of genetic alterations in the literature, such as “*differential expression*” of a gene and gene “*splice variants*”, leading to greatly extended curation results of cancer genetic alterations.

Despite significant progress, there is still big room for the comprehensive interpretation of cancer pathology. For example, the Hallmarks of Cancer continue to evolve^19^. In the 2022 update^4^, the framework expanded to encompass 14 hallmark features. Nevertheless, there remains value in investigating additional comprehensive biological processes and clinical phenotypes within the context of cancer pathology. Furthermore, it is a trend to shift the focus on genetic alterations from standard point mutations to a broader range of general genetic alterations. However, the study in the investigation of general alterations with the molecular mechanism is sparse. Therefore, there is a growing demand to integrate datasets and link cancer biomarkers with underlying molecular mechanisms. The availability of such databases is capable of advancing the understanding of cancer pathology.

In response to the aforementioned concerns, we propose Cancer-Alterome, a literature-mined dataset. This dataset focuses on the identification and extraction of the following.

- Diverse genetic alterations: It captures various types of genetic alterations, going beyond standard point mutations, to encompass a broader range of general genetic alterations.
- Expanded hallmarks of cancer: The dataset seeks to capture and analyze not only well-established hallmarks but also novel biological processes or clinical phenotypes associated with cancer, as reported in the literature.
- Genetic alteration regulatory event (GARE): A GARE is a regulatory event caused by genetic alterations, and it includes the resulting biological process or clinical phenotype for the interpretation of cancer pathology.

This dataset is established through the application of a series of text-mining tools and methodologies. The pipeline first identifies and extracts SNPs, DNA changes, and protein changes and normalizes them to unique identifiers in dbSNPs. In addition, it incorporates the dictionary adopted in CIViC^18^, extracts mentions of epigenetic changes, expression changes, and structural variations and classifies them into distinct categories. Subsequently, the pipeline extracts a broader range of biological processes and clinical phenotypes in literature. These terms are further normalized into concepts in Gene Ontology (GO), Human Phenotype Ontology (HPO), and Medical Subject Headings (MeSH), allowing extensive investigations of cancer pathology. Three distinct ontologies interpret mechanisms across different facets, although there is no absolute separation in their functions. GO is primarily employed to elucidate molecular mechanisms, HPO serves to provide phenotype descriptions following alterations, and MeSH is primarily utilized to offer a unified definition at the disease level. A template-matching strategy is then applied to filter GARE events. We evaluate the performance of each text mining tool and compare its soundness with a large language model (LLM). Finally, the pipeline extracts 13,259K GARE records from a mass literature database with 4,354K PubMed abstracts and PMC full texts that are related to pan-cancer topics. The GARE records cover 21K genes, 157K genetic alterations, and 154K downstream biological processes and clinical phenotypes.

Additionally, case studies are conducted to assess the data reusability of Cancer-Alterome. All cases indicate that the dataset facilitates the multifaceted investigations of cancer pathology from specific genes or cancers. The resulting dataset largely facilitates the interpretations and traceability of cancer pathology, enabling valuable insights into evidence-based precision oncology. A web service is established, allowing gene-specific, cancer-specific, and gene-set queries upon the dataset. In response to the queries, the web service provides intuitive statistical diagrams and easy-to-read evidence tables.

## Methods

**Fig. 1:**
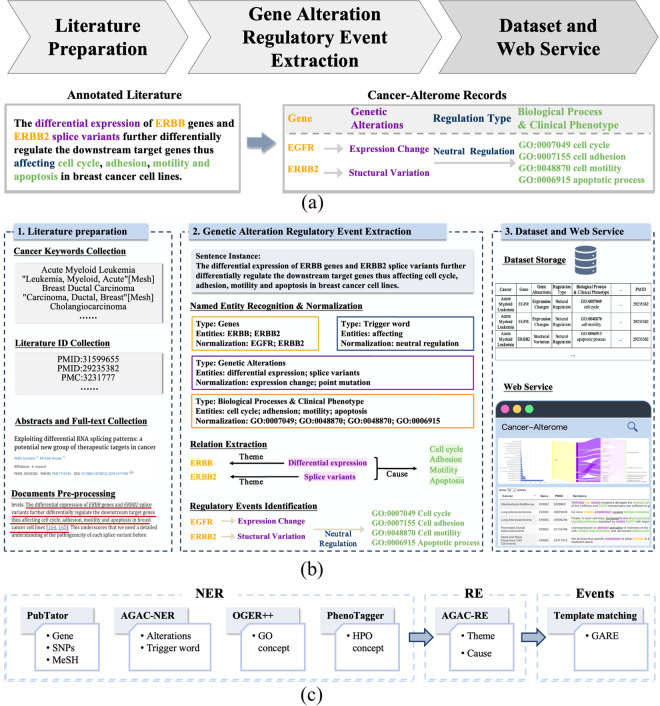
Data mining pipeline of Cancer-Alterome. (**a**) Overview of the pipeline. (**b**) Data processing details of the pipeline with an example. (**c**) NLP tools and methods used in the GARE extraction.

We construct a text-mining pipeline for Cancer-Alterome. The pipeline comprises three parts (Fig. 1a): literature preparation, genetic alteration regulatory event extraction, and dataset and web service. The annotated literature is then transformed into database records, which serve to present regulatory events caused by genetic alteration. The detailed information on the pipeline is as follows.

### Literature preparation

The literature preparation part involves the retrieval and filtering of all cancer-relevant abstracts and full texts. As illustrated in Fig. 1b, there needs four steps in data preparation.

#### Cancer keywords collection

Cancer-Alterome focuses on 32 pan-cancers defined in the database of The Cancer Genome Atlas (TGCA, https://www.cancer.gov/ccg/research/genome-sequencing/tcga/studied-cancers). To retrieve the literature from PubMed and PMC, disease terms (e.g. *Acute Myeloid Leukemia*) and corresponding MeSH terms (e.g. *“Leukemia, Myeloid, Acute”[Mesh]*) of 32 cancers are manually curated and set as keywords for the literature searching. To avoid mismatching, the abbreviations of cancer names (e.g. *AML*) are excluded from the keywords list.

#### Literature ID collection

After the keywords preparation, the Esearch and Efetch, standard tools in NCBI Entrez Direct toolkit for resource download, are employed to perform literature searching. Specifically, the command *“esearch -db pubmed -query ‘Acute Myeloid Leukemia’ | efetch -format uid”* are utilized for PubMed retrieval, while the commend *“esearch -db pmc -query ‘Acute Myeloid Leukemia’ | efetch -format uid”* are used for PMC retrieval. The retrieved PubMed identifiers (PMID) and PMC identifiers (PMCID) are stored.

#### Abstracts and full texts collection

The pipeline utilizes a NCBI-released data retrieval API, PubTator API^20^, to retrieve documents by providing the PMIDs and PMCIDs. The API returns regularized links, which were processed using the Python packages “Request” and “BeautifulSoup” to request and parse the content. Subsequently, the document, along with the literature information (e.g., journal and publication year), is saved in a BioC-JSON format file.

#### Documents pre-processing

Documents pre-processing is performed after literature downloading. First, the pipeline filters the documents by keyword matching. Literature remains only when full names or abbreviations of cancer are mentioned at least three times in the abstract or five times in the full text. Second, the pipeline splits the document into individual sentences using the Python package “NLTK” and “spaCy”. Subsequently, it removes the sentence with incorrect format parsing.

### Genetic alteration regulatory event extraction

After the literature preparation, the pipeline further identifies and normalizes the biological entities mentioned in the literature and extracts the relations between these entities. This three-step process leverages multiple text mining tools and methodologies with high quality, yielding precise GARE extractions in literature. For example, we mainly use it to tag genes, point mutations, and MeSH terms in texts, as shown in Fig. 1c. Actually, PubTator^20^ has long been a top-rated tool^21^ enabling large-scale annotation of genes, mutations, and ontologies like MeSH. Furthermore, OGER++^22^, PhenoTagger^23^ and AGAC-NER^24^ are found to be top-rated NER tool in tagging GO terms, HPO terms and genetic alterations^24–26^.

#### Named entity recognition and normalization

In this step, multiple established tools are employed to identify biological entities mentioned in the sentences, and then normalize them into unique identifiers in the database. First, PubTator API is used to annotate the mentions of genes and proteins in the literature and normalize them to NCBI gene ID. It is also used to extract point mutations and normalize them to corresponding dbSNP IDs by using tmVar3. Additionally, OGER++^22^ and PhenoTagger^23^ are utilized to identify the mentions of biological processes and clinical phenotypes, and then normalize these entities into GO and HPO concepts, respectively. By fine-tuning a deep neural network combining BioBERT embedding and CRF decision layer, we apply AGAC-NER^24^ to extract the genetic alterations and trigger words from the text. Utilizing the same training dataset and fine-tuning strategy as employed in AGAC-NER^24^, we replicate the entity extraction strategy pertaining to the genetic alterations and trigger words, achieving the planned entity recognition objectives. Taking a sentence example in Fig. 1b as an example, AGAC-NER captures the description of genetic alterations, and links “*the differential expression of ERBB*” and “*ERBB2 splice variants*”. Furthermore, it identifies the trigger words in the regulatory event, such as “induce”, “promote” and “inhibit”. These trigger words are further categorized into three regulation types, i.e., neutral regulation, positive regulation, and negative regulation. To avoid labeling conflicts, in the case when multiple entities overlap, only the entity with the longest span is retained.

**Fig. 2:**
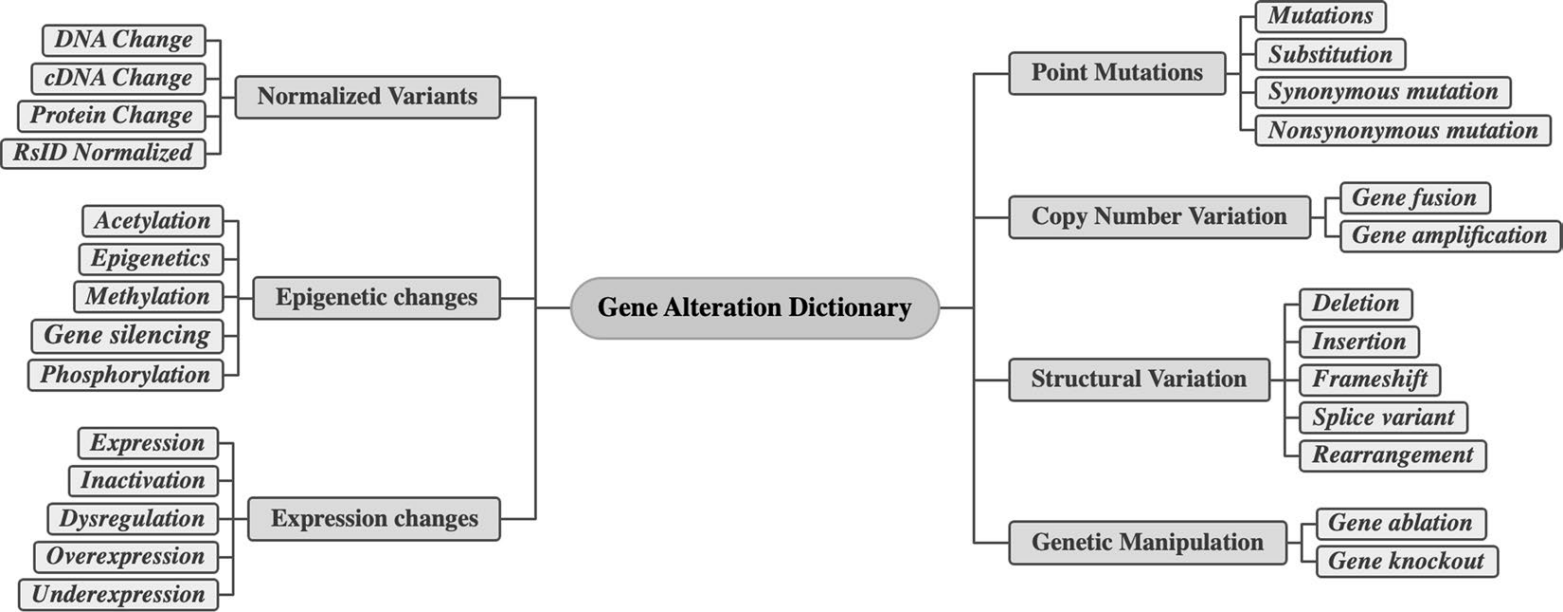
Structure of genetic alteration dictionary.

Cancer-Alterome takes extensive steps in mutation and alteration curation. First, tmVar3^27^ is used to identify and extract SNPs, DNA changes, and protein changes and normalize them to unique identifiers in dbSNPs. For genetic alterations, we adopt the dictionary of CIViCmine^18^, which includes common mutations, aberrations, and other omic events that may occur to a gene, especially in the cancer setting. To expand the dictionary, the Top500 most frequently mentioned genetic alterations are added as synonyms in specific categories of genetic alterations. As shown in Fig. 2, the curated genetic alteration dictionary contains 7 secondary categories and 27 tertiary categories. The updated dictionary merges similar categories in the original dictionary and significantly increases the number of synonyms for each category (Table 1). Compared to CIViCmine, the synonym count increases from 455 to 549 in the updated dictionary.

**Table 1:** Comparison of dictionary statistics in Cancer-Alterome and CIViCmine.

Genetic alterations Dictionary	* in CIViCmine^18^	* in Cancer-Alterome
# of categories	40	27
# of synonyms	455	549
# of average synonyms	11	20

#### Relation extraction

Following the NER step, a relation extraction step is performed by AGAC-RE^24^ to identify two types of relations, i.e., *Theme* and *Cause*. The *Theme* relation indicates a genetic alteration *occurs in* a specific gene, while the *Cause* relation indicates a genetic alteration *causes* a downstream biological process or clinical phenotypes. We again take the sentence in Fig. 1b as an example, the RE computation processes the sentence “*differential expression of ERBB genes...affecting cell cycle*” and outputs that *differential expression* plays a semantic *Theme* role on *ERBB*, and a semantic *Cause* role on *cell cycle*. It forms an event that *a differential expression occurs in ERBB and causes cell cycle*. In the RE step, all alteration entities included in the relation are calibrated by the dbSNP database and the updated alteration dictionary. Specifically, the extraction of relations in AGAC-RE is predicated upon the foundation of joint learning with AGAC-NER. The two models share a weighted-sum loss function. Through the optimization of this loss function via joint learning, the most optimal results are achieved on the training dataset.

#### Regulatory events identification

Regulatory events are identified by applying a rigorous template-matching strategy. The template defines how a genetic alteration *occurs in* a specific gene and how it subsequently *causes* the downstream biological processes and clinical phenotypes.

1. Only sentences containing gene, genetic alterations, downstream effect, *Theme* relation, and one *Cause* relation are filtered.
2. The regulation types are classified into *neutral regulation*, *positive regulation* and *negative regulation*, based on the trigger words type.
3. Collect RE result of “*Gene* $\mathop{\leftarrow }\limits_{Theme}$ *Mutation*”, and “*Mutation* $\mathop{\to }\limits_{Cause}$ *biological process or clinical phenotype*.”
4. Sentences containing the demanded entities and identified regulation type are mapped into the corresponding regulatory event, i.e., “*Gene* – *Mutation* – *Regulation* – *Biological process or clinical phenotype*.”

We again use the sentence example in Fig. 1b,and eight regulatory events are extracted from the sentence instance. The record, “*EGFR* –*Expression Change* – *Neutral Regulation* – *GO:00040790 cell cycle*”, is one of the obtained standardized events from the GARE identification process.

### Dataset and web service

We establish the Cancer-Alterome dataset with all the extracted GARE records. Each record in the dataset is then linked with corresponding literature support for evidence-tracing. Sentences carrying regulatory events are visualized in a user-friendly web service, http://lit-evi.hzau.edu.cn/PanCancer. It offers quick evidence query by single gene query, gene set query, single cancer query, or cancer set query. The web service also provides illuminative investigation of regulatory events with heatmap and statistical tables. Benefiting from comprehensive annotations of cancer pathology elements, Cancer-Alterome exhibits extensive potential usage for data exploration.

#### Visualize the regulatory events caused by genetic alteration

**Fig. 3:**
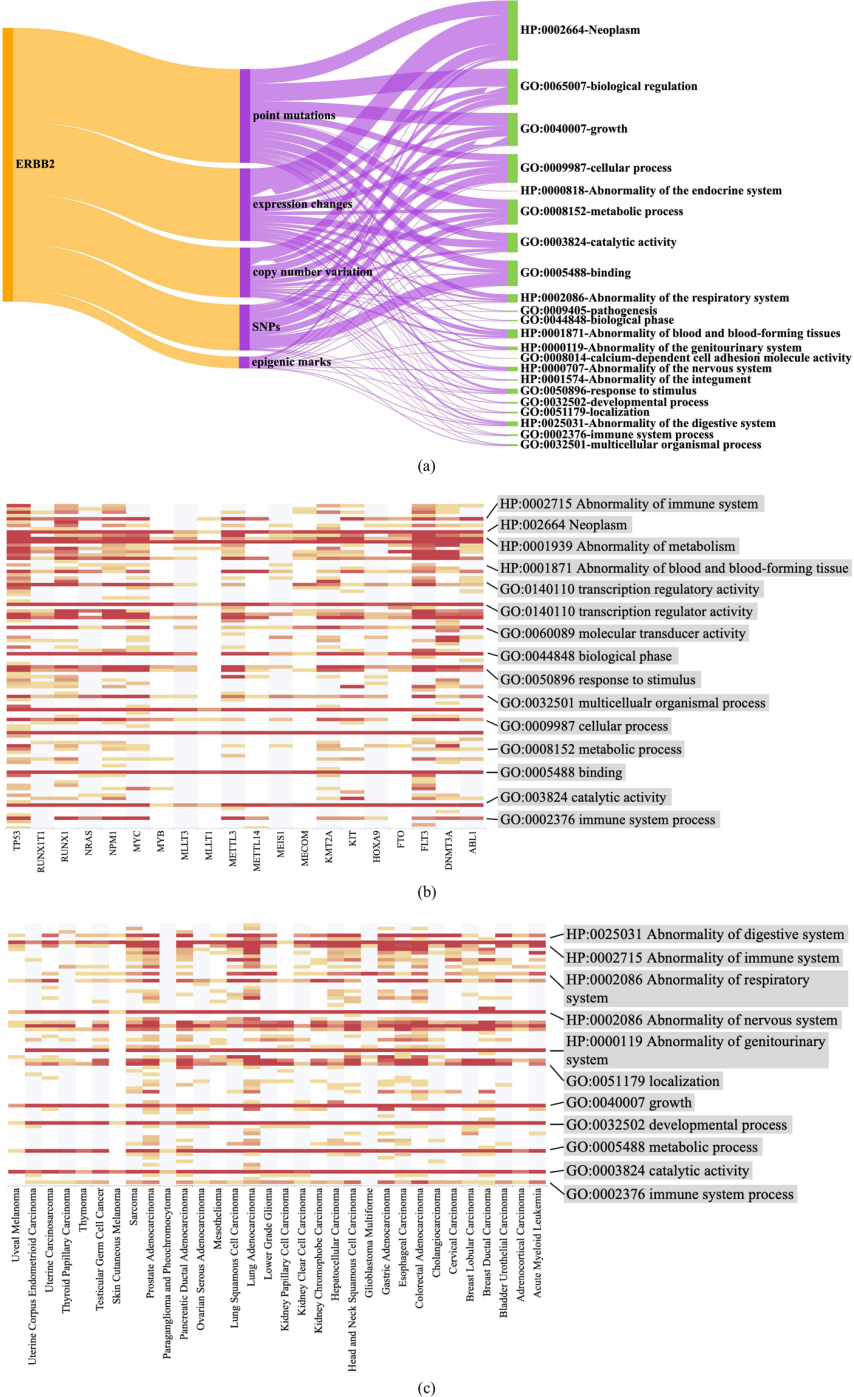
Usage notes of the Cancer-Alterome resource. (**a**) Sankey diagram of regulatory events caused by ERBB2-related alterations. (**b**) Heatmap of top-20 AML-related genes with regulatory events in Cancer-Alterome. (**c**) Heatmap of ERBB2 and associated events in 32 pan-cancers.

We select the well-known cancer driver gene ERBB2 and check the genetic alterations related to this gene within the context of AML in the dataset. As shown in Fig. 3, diverse genetic alterations are linked with ERBB2, including expression changes, point mutations, copy number variation, etc, as represented in Fig. 3. These genetic alterations are further linked to the regulation of downstream biological processes or clinical phenotypes, clustered with relevant GO and HPO ancestors such as HP:002664-Neoplasm, GO:0065007-biological regulation, and GO:0040007-growth. A portion of these terms pertain to the defined cancer hallmarks. For instance, GO:0008152 metabolic process corresponds to “reprogramming cellular metabolism”, GO:0002376 immune system process corresponds to “avoiding immune destruction”. Meanwhile, other specific cancer hallmark terms can be found in the records of the child nodes of these GO, HPO ancestors. For example, GO:0006915-apoptotic process is categorized as GO:0009987-cellular process, and GO:0006954-inflammatory response is categorized as GO:0050896-response to stimulus. In addition to these widely used cancer hallmarks, the Sankey diagram also suggests some other known GO terms, such as GO:0001871 Abnormality of blood and blood-forming tissues, and GO:0000707 Abnormality of the nervous system. These terms illustrate specific pathological mechanisms for ERBB2 in specific cancers.

#### Pathology investigation

Cancer-Alterome can be utilized to investigate cancer pathology from a broader perspective. Figure 3b represented the heatmap of top-20 AML-related genes from Cancer-alterome to the downstream effects, facilitating investigation of the pathological mechanisms involving multiple genes for a specific cancer. Heatmap in Fig. 3c illustrates the associations cluster of ERBB2 with downstream effects across 32 pan-cancers. Multiple significant clusters can be observed. BP clusters, e.g., GO:0032501 multicellular organismal process and GO:0000187 immune system process, suggest common mechanisms of ERBB2. In the meantime, phenotype clusters, including HP:0025031 Abnormality of digestive system and HP:0000119 Abnormality of the genitourinary system, indicate the clinical performance related to ERBB2. The heatmap facilitates the association of genes with known cancer hallmarks. For instance, a hallmark of “avoiding immune destruction” is significantly clustered in ERBB2 records. In conclusion, by leveraging Cancer-Alterome, we can explore the pathological mechanisms of cancer from multiple perspectives. Specifically, we can investigate the downstream effects of genetic alterations in specific genes, as well as the functional enrichment of multiple genes in different cancers. Moreover, in the context of expanding cancer hallmarks, the dataset allows for a broader perspective in interpreting cancer pathology. Notably, all these associations can be further traced back to literature support, greatly contributing to the interpretability of cancer pathology. Overall, Cancer-Alterome has the potential to be a significant valuable resource for precision oncology and cancer pathology research.

#### Web service construction

To improve data accessibility, we have developed a user-friendly web service accessible via http://lit-evi.hzau.edu.cn/PanCancer. This platform encompasses a range of interactive pages designed to facilitate seamless exploration and utilization of the dataset:

- By Gene: On this page, users have the option to query a single gene and receive comprehensive reports for the queried gene across 32 pan-cancers. The reports include information about genetic alterations specific to each cancer type, as well as the regulatory events associated with these alterations. Moreover, the platform features an interactive rich-text presentation table located below the statistical graphs. This table serves to enhance the user’s experience by providing an easy-to-navigate interface for accessing literature support related to the queried gene and its associated genetic alterations and regulatory events.
- By Cancer: This page, similar to the “By Gene” page, allows users to query a single cancer and investigate the statistical diagram and literature support of the cancer.
- By Gene Set: This page enables users to enter a gene set, and investigate the clustering of cancers related to those genes in the expanded cancer hallmarks. The results will be represented as a visualized heatmap and an interactive table.
- By Cancer Set: This page, similar to the “By Gene Set” page, enables users to input a collection of cancers, analyze the clustering of genes linked with those cancers with the expanded cancer hallmarks, and depict the results through a visual heatmap and an interactive table.

Through the web service, data downloading is allowed for each query, and an option is also given to download the complete dataset. Additionally, to ensure continuous data availability, the information within the web service will be updated on a quarterly basis.

## Data Records

The dataset is available at Figshare^28^. To facilitate scalable maintenance and convenient data access, we present two distinct data formats, SQLite 3 and tab-separated values (TSV).

SQLite3 format is lightweight. This format enables swift data importation into downstream applications, including the Django framework, and can be effortlessly converted into a MySQL database. As a result, direct database querying becomes feasible and efficient.

TSV files provide easy-to-view access to data. This file encompasses 13,256K rows of Cancer-Alterome records, structured into 21 columns denoted from A to V. Below are the specific descriptions for each column:

A. Cancer: The full name of the cancer for one of the 32 pan-cancers, e.g. “*Acute Myeloid Leukemia*”.
B. Gene: The NCBI gene symbol of the gene reported in the records, e.g. “*EGFR*”.
C. EntrezID: The NCBI Gene ID for the gene in column B, e.g. “1956”.
D. Normalized Variants Mention: Standard dbSNP or normalization for other genetic alterations are listed, including the mentions in the text, e.g. “*L858R*”. In cases where the normalized term is absent or unavailable, this column is indicated by a dash symbol (“-“).
E. Normalized Variants ID: The dbSNP identifier of genetic variant in column D, e.g. “*rs121434569*” or “*g.4717C>G*”.
F. Normalized Variants Type: Four types of normalized variants, including rsID, DNA change, cDNA change, and protein change.
G. Alteration Mentions: The mention of genetic alteration in the record. In Fig. 1b, examples of such mentions include “*differential expression*” and “*splice variants*”.
H. Alteration Type: The 7 types and 27 sub-types of the genetic alterations, as defined in Fig. 2. For example, the above two examples in column G fall into alteration types “*Expression Change:Expression*” and “*Structural Variation:Splice variant*”, respectively.
I. GO Mention: The mentions of the GO concepts in this records, e.g. “*cell cycle*” or “*adhesion*” in Fig. 1.
J. GO Term: The normalized GO ID and concept of the GO mention in column I, e.g. “*GO:0007049 cell cycle*” or “*GO:0007155 cell adhesion*”.
K. GO Ancestor: The ancestor node at the second level in the ontology of the GO term in the column J. For example, “*GO:0065007 biological regulation*” is the ancestor node of “*GO:0042129 regulation of T cell proliferation*”.
L. HPO Mention: The mentions of the HPO concepts in the records, e.g. “*decreased immune response*”.
M. HPO Term: Normalized HPO ID and name of the HPO mention in column M, e.g. “*HP:0002721 Immunodeficiency*”.
N. HPO Ancestor: The ancestor node of the HPO term in column M, e.g. “*HP:0002715 Abnormality of the immune system*”.
O. MeSH Mention: The mentions of the MeSH concepts in the record, e.g. “*autoimmune diseases*”.
P. Mesh Term: Normalized MeSH ID and name of the MeSH mention in the column O, e.g. “*MeSH:D001327 Autoiummune Diseases*”.
Q. Include Event: The “true” or “false” indicates whether the record contains a complete description of the regulatory events.
R. Regulatory Events: The complete description of the regulatory events in the record, e.g. “*EGFR --Expression Change -- Neutral Regulation -- GO:00040790 cell cycle*” (example in Fig. 1b).
S. PMID: PMID of the source literature.
T. Sentence: The sentence instance linked with the record.
U. Journal: Name of the journal in which the source literature was published.
V. Year: Publication year of the source literature.

## Technical Validation

### Checking for accuracy of the data mining pipeline

#### Accuracy evaluation of text mining tools and methodology

**Fig. 4:**
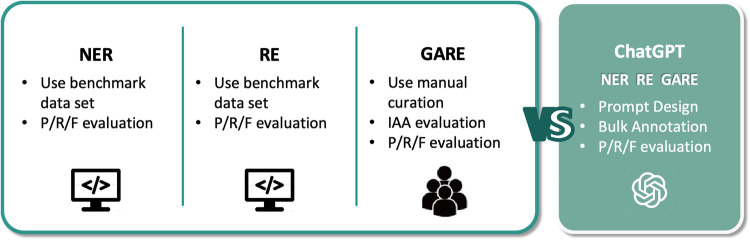
Evaluation steps for text mining tools and methodology.

To guarantee the precision of the extraction results, a series of well-established text mining tools are utilized within the pipeline. As shown in Fig. 4, we take extensive evaluations upon text mining tools and methodology used in NER, RE, and GARE extraction. Benchmark data are used for NER and RE evaluation, while manual curation is applied for GARE evaluation. Precision (P), recall (R), and F1-score (F) are taken as evaluation metrics in all the settings. In addition, inter-agreement annotation (IAA) is adopted in all the manual checks.

The performance evaluation of NER, RE, and GARE extraction are presented in Table 2, respectively. In brief, model performances of PubTator, OGER++, and PhenoTagger are reported in the original paper. AGAC-NER and AGAC-RE are evaluated in CHIP 2022 open shared tasks^29^. The quality of GARE events is manually evaluated, as detailed below.

**Table 2:** Accuracy evaluation of literature mined results in Cancer-Alterome.

Named entity recognition and normalization		Evaluation metric			Count of prediction		
Tools	Entity type	Precision	Recall	F1 Score	Mention	Entity	Normalization
PubTator^20^	Gene	0.79	0.81	0.80	203,939	22,021	Entrez ID
PubTator^20^	Point mutations and SNPs	0.81	0.81	0.83	197,595	164,144	rsID
AGAC-NER^24^	General genetic alteration	0.74	0.57	0.64	3,002,082	549	Dictionary
AGAC-NER^24^	Trigger word	0.78	0.70	0.74	200,670	—	—
OGER++^22^	GO	0.72	0.17	0.27	52,140	22,874	GO ID
PhenoTagger^23^	HPO	0.79	0.70	0.74	24,261	8,345	HPO ID
PubTator^20^	MeSH	0.83	0.82	0.81	1,131,933	45,119	MeSH ID
**Relation extraction**		**Evaluation metric**			**Count of prediction**		
**Tools**	**Relation type**	**Precision**	**Recall**	**F1 Score**	**Relations**		
AGAC-RE^24^	*Theme*	0.87	0.84	0.91	37,411,752		
AGAC-RE^24^	*Cause*	0.88	0.85	0.82	12,420,489		
**Regulatory events identification**		**Evaluation metric**			**Count of prediction**		
**Method**	**Event type**	**Precision**	**Recall**	**F1 Score**	**Events**		
Template match	GARE	0.84	0.96	0.90	16,681,473		

(a) Evaluation of NLP tools for named entity recognition and normalization. (b) Evaluation of NLP tools for relation extraction. (c) Evaluation of regulatory events (GAREs).

Performance results shown in Table 2 suggest that the adopted tools exhibit high precision in NER. For instance, OGER++ achieves the lowest precision of 0.72 in recognizing GO terms, and PubTator gets the highest precision of 0.81 for recognizing both genetic variations and Mesh terms. Though OGER++ obtains a lower recall rate for GO extraction at 0.17, it is attributed to the inherent complexity of the task involving GO identification and normalization. Meanwhile, low recall is acceptable for the dataset construction, since the accuracy holds greater significance for the integrity and utility of the dataset. After the NER step, abundant entities are recognized and normalized upon a mass literature database with 4,354K PubMed abstracts and PMC full texts. For example, the pipeline captures 203,939 gene mentions. After normalization by Entrez ID, these mentions correspond to 22,021 unique gene entities. The full statistics are given in the table.

Moreover, performance results in Table 2 suggest that AGAC-RE demonstrates commendable precision and recall in the RE task, indicating the high accuracy of the extracted result. Upon sentences annotated by the NER task, we perform AGAC-RE to derive relations. In total, the pipeline captures 37,411,752 *Theme* relations and 12,420,489 *Cause* relations from the filtered literature.

Subsequently, we then apply the template-matching strategy to the obtained sentences containing the above entities and relations and filter GAREs accordingly. To evaluate the accuracy of the generated GAREs, 2000 randomly selected GAREs are manually checked by four human experts from the fields of biology, bioinformatics, and biomedical natural language processing. Rigorous rules are applied, requiring the records should carry fully correct biological event identification, including the gene, genetic alteration, and the downstream effects. The detailed manual check procedure, IAA result, and performance evaluation are presented in Section 1, Supplementary. Eventually, a high F-score of 0.90 is obtained after the evaluation, indicating the quality of the obtained regulatory events. Application of the template-matching strategy yields abundant GAREs. In total, 16,681,473 GAREs are curated finally.

#### Analysis of the possible impacts caused by cascade error in the GARE extraction pipeline

Generally, the evaluation of the GARE extraction is mainly based on the outcome of the regulatory events in the final step, as suggested with an F1-score of 0.90 (Table 2). In addition to the fundamental evaluation, it should be noted that GARE extraction is performed in a workflow manner, and the performance of data processing and prediction in each step may have a potential impact to affect the outcome of GARE extraction. To fully address these considerations, we, therefore, analyze the possible errors cascaded in the data-processing process, NER step, and RE step, and conclude multiple impacts or effects. In brief, sentence splitting as an example of text pre-processing has won good quality, and Fisher’s exact test shows that the wrongly split sentence will not significantly affect the GARE extraction. Furthermore, missing NER tagging will bring silence in RE and GARE extraction. Incorrect NER results will bring the noise in RE and may bring the noise to GARE. In addition, correct NER and incorrect RE results may bring the noise to GARE extraction. Wrong trigger word recognition, mainly comes from the wrong tagging of negation, which also brings the noise in GARE. The full analysis is given in Section 2, Supplementary.

#### The potential improvements by leveraging large language model (LLM)

Moreover, concerning the potential advancements achieved by the LLM-based method in our task, we take prompt engineering with ChatGPT and test its performance in NER, RE, and GARE extraction tasks. An overall F1-score comparison on GARE extraction, i.e., 0.90 vs. 0.57 for the proposed pipeline and LLM-based method, indicates that the proposed pipeline presents competitive performances if compared with the LLM result.

For additional details, we utilize the gpt-3.5-turbo model as LLM and perform prompt engineering to replicate NER, RE, and GARE extraction tasks listed in Table 2, employing the same datasets as outlined in the original tool papers. Specifically, PubTator performs gene, disease, and mutation tasks by utilizing the BioCreative II GN^30^, NCBI Disease^31^, and BRONCO^32^ data, while AGAC-NER and -RE perform employ the AGAC corpus^33^. In addition, the evaluation of OGER and PhenoTagger utilize the CRAFT Corpus^34^ and GSC+ corpus^35^, respectively. Eventually, GARE evaluation is conducted using manually annotated gold standard datasets. Very recently, Chen *et al*.^36^, publicize the comparison result in NER on disease and gene with PubTator and ChatGPT. In the meantime, Labb*é et al*.^37^, compare PhenoTagger with ChatGPT in NER on HPO terms. With these advancements, we therefore design task-specific prompts for the remaining 7 tasks. All prompts and evaluation scripts are available in the https://github.com/bionlp-hzau/Cancer-AlteromeGitHub repository, https://github.com/bionlp-hzau/Cancer-Alterome. The detailed experimental setting and comparison results are given in Section 3, Supplementary. In almost all tests, the ChatGPT-based approach does not outperform the selected model, consistent with recent evaluations in biomedical text recognition^38^. It seems that due to the domain-specific terminology in biomedical texts, current LLMs are still falling short in tasks such as NER and RE, despite often excelling in context-relied tasks like question answering (QA). However, considering that the prompt engineering test represents only our attempt in this research, along with the ongoing evolution of LLMs, the obtained results primarily serve as supporting evidence for the reliability of the Cancer-Alterome outcomes.

### Checking for data abundance of the database

#### Counts of biomarkers, biological processes, and clinical phenotypes

As stated in the last section, Cancer-Alterome encompasses 16,681,473 regulatory events, with each cancer containing an average of 521K regulatory events. These records contain a comprehensive set of annotations for biomarker entities, including the associations between 21K genes and 136K normalized genetic variations, plus 20K genetic alterations (Table 3). The dataset also collects a large number of associations between these biomarkers and the expanded cancer hallmarks, including over 4K GO terms, 2K HPO terms, and 146K MeSH terms.

**Table 3:** Unique entity statistics in Cancer-Alterome.

Biomarker entities, biological processes and clinical phenotypes	Unique Count	Average count per cancer
Gene	21,997	11,181
Normalized point mutations and SNPs	136,163	14,432
General Genetic Alterations	20,886	1,848
GO concept	4,733	1,258
HPO concept	2,989	458
MeSH concept	146,932	19,556

**Fig. 5:**
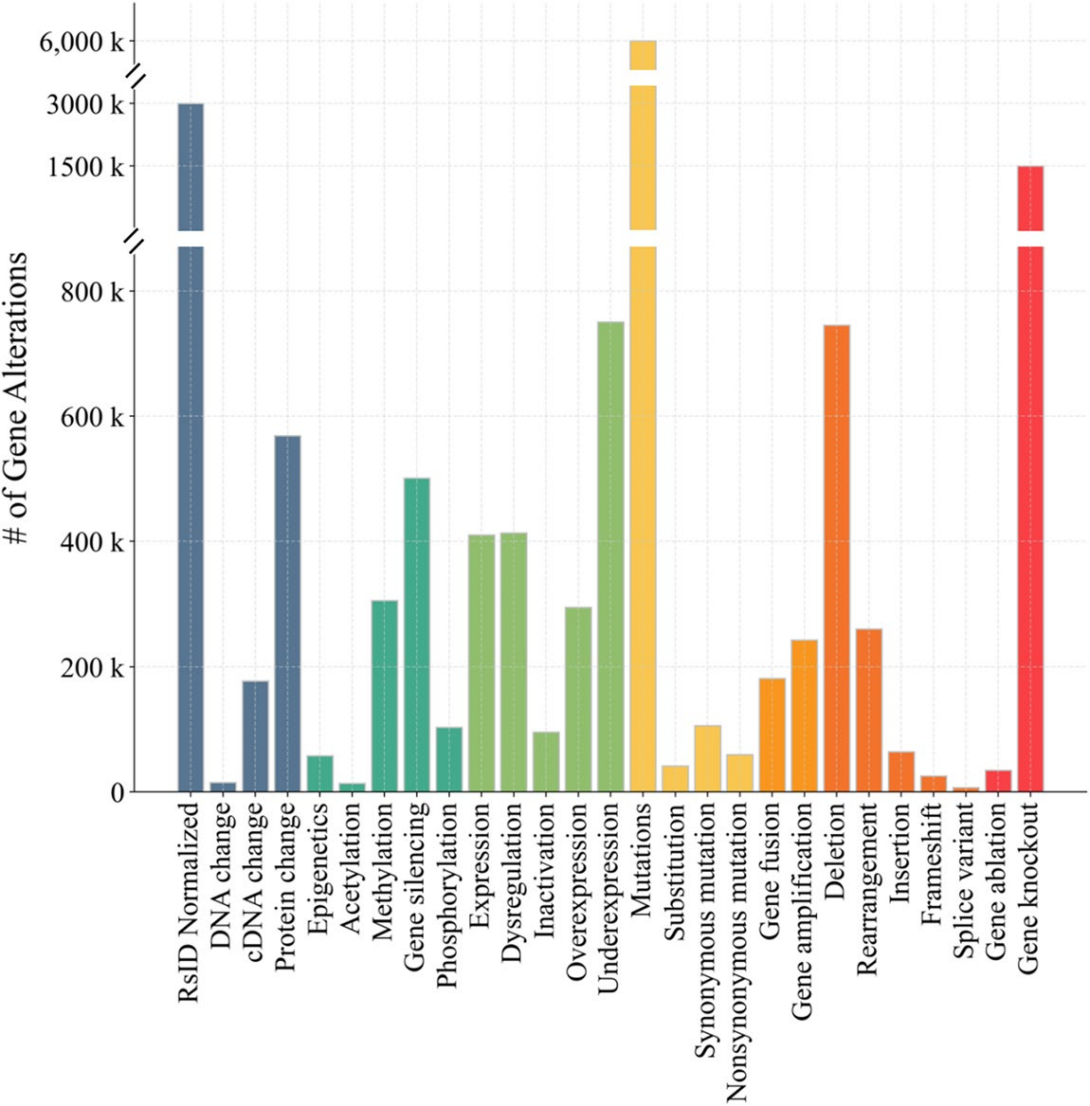
Counts of genetic alterations defined in Cancer-Alterome for pan-cancers.

Focusing on the extensive statistical result of the genetic alterations, we investigate the coverage of 27 types of genetic alterations in the dataset. As shown in Fig. 5, “Mutations” are mentioned most significantly in the dataset, followed by rsID normalized variations. Multiple genetic alterations, such as “Acetylation” and “Splice variant” are comparatively less prevalent in the dataset, possibly owing to lower reporting frequency in cancer research. Notably, all the GARE records include the sentence support and literature information, providing metadata for the web service in this research.

#### Yearly statistics for the count of regulatory events recorded per cancer type

**Fig. 6:**
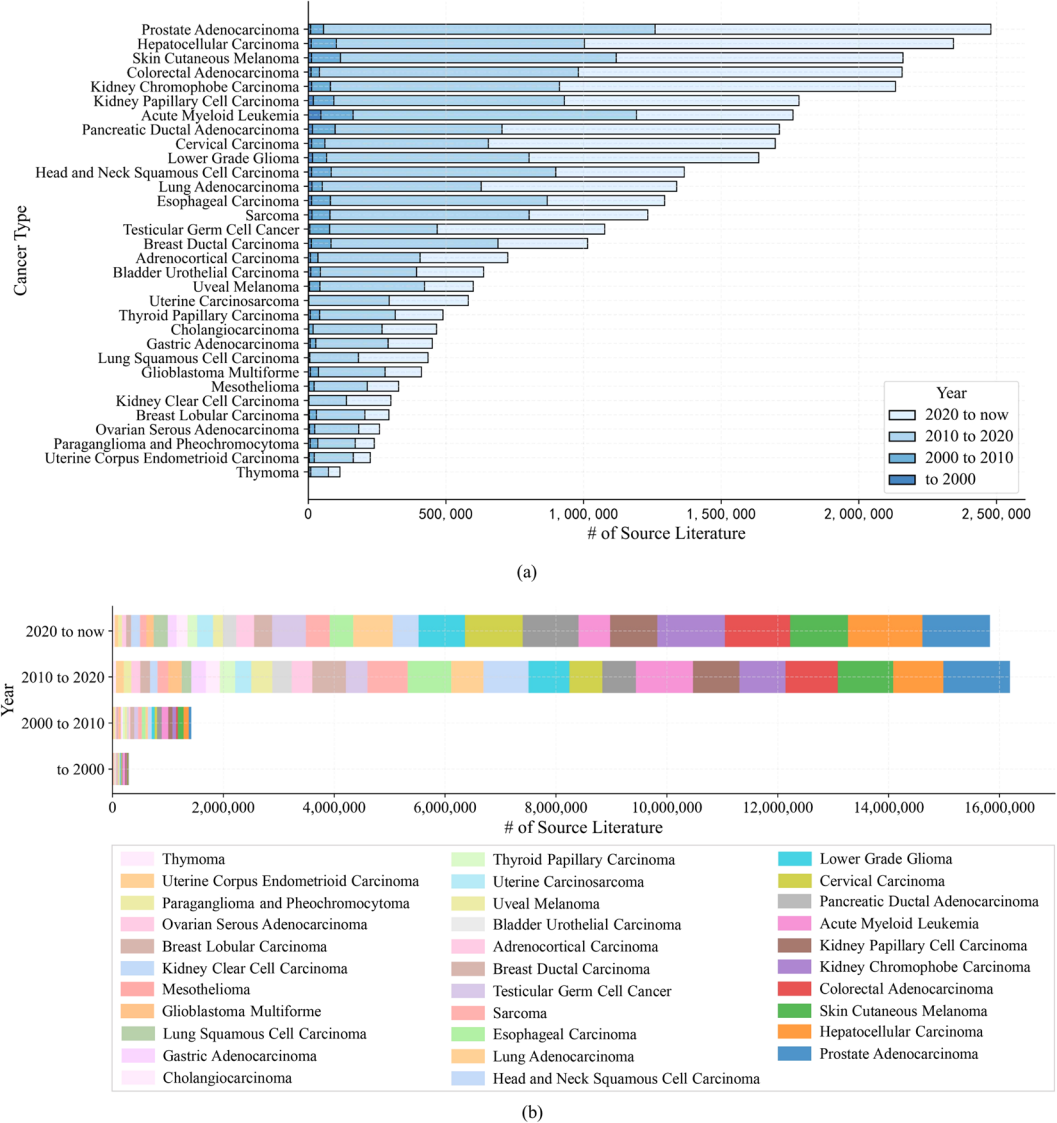
Yearly distribution of genetic alteration regulatory events across 32 pan-cancers on a yearly basis. (**a**) Distributions based on cancer types. (**b**) Distributions based on year periods.

We plot the distribution of regulatory events with publication year across four distinct time periods for 32 cancers (Fig. 6a). The high coverage of specific cancers such as “Prostate Adenocarcinoma” and “Hepatocellular Carcinoma” highlights the significant areas of focus and prevailing trends within the field of cancer research. It can be observed that there has been a substantial surge in research literature about the extracted regulatory events, particularly after the year 2010. Remarkably, in just three years from 2020 to the present, the amount of extracted regulatory events has surpassed that of the previous decades, demonstrating an impressive growth trend (Fig. 6b).

#### Comparing data abundance across benchmark databases

For the external evaluation of the dataset abundance, we compare Cancer-Alterome with other multiple literature-mining-based data resources for cancer. These resources include CancerMine^17^ for cancer genes and CIViCmine^18^ for genetic alteration in cancers. We select six representative genes from CancerMine, which include three most frequently reported genes in the context of acute myeloid leukemia (AML), KMT2A, FLT3, and MLLT3, as well as three genes that are least reported in this specific cancer, namely ZFP36L2, ZIC2, and ZNF582. Table 4 indicates that Cancer-Alterome presents a greater number of records for all six genes compared to CancerMine and CIViCmine. Furthermore, CancerMine and CIViCmine focus on only single biomarkers, genes, or genetic variants in precision oncology. Cancer-Alterome goes beyond these limitations. It not only widens the spectrum of encompassed genetic alterations but also establishes intricate correlations with downstream effects, thereby elevating the systematic interpretation of cancer pathology.

**Table 4:** Comparison of data abundance across benchmark resources.

Resource	Biomarkers and phenotype			Literature support	Count of database records w.r.t exampled gene queries					
	Gene	Alteration	Event		KMT2A	FLT3	MLLT3	ZFP36L2	ZIC2	ZNF582
Cancer-Alterome	✓	✓	✓	✓	7,322	25,946	497	9	29	7
CancerMine	✓	NA	NA	✓	206	154	151	1	1	1
CIViCmine	✓	✓	NA	NA	80	571	15	0	0	0
GePI	✓	NA	✓	✓	8,322	7,965	1,156	309	609	48

Furthermore, we conduct a comparison of Cancer-Alterome with existing databases that report biological events. Since the known resources, like DigSee^9^ and BioContext^8^, are currently not available, we focus the statistical comparisons solely on GePI. In GePI, the record number of these six genes under full diseases context is 8,322, 7,965, 1,156, 309, 609, and 48, respectively, having a comparable size with our dataset. While, these three databases only focus on seven categories of downstream biological events, including gene expression, transcription, phosphorylation, localization, regulation, binding, and protein metabolism. The expanded cancer hallmarks in our dataset cover all concepts in GO, HPO, and MeSH, thus enhancing the interpretation of cancer pathology.

### Supplementary information

Supplementary file

## Data Availability

The scripts utilized to parse literature and extract events are home-written codes which are publicly available at GitHub repository https://github.com/bionlp-hzau/Cancer-Alterome. The underlying python3 libraries used in this project are all open-source: E-direct (https://ftp.ncbi.nlm.nih.gov/entrez/entrezdirect), OGER++ (https://github.com/OntoGene/OGER), PhenoTagger (https://github.com/ncbi-nlp/PhenoTagger), PubTator (https://www.ncbi.nlm.nih.gov/research/pubtator/), AGAC-based model (AGAC-NER: https://github.com/YaoXinZhi/BERT-CRF-for-BioNLP-OST2019-AGAC-Task1 and AGAC-RE: https://github.com/YaoXinZhi/BERT-for-BioNLP-OST2019-AGAC-Task2), Pytorch (http://www.pytorch.org) and sci-kit-learn (http://scikit-learn.ory). More details on the guidelines of code usage are given in Supplementary 4.
