# Supplementary material for: Cancer-Alterome: a literature-mined resource for regulatory events caused by genetic alterations in cancer
Source: Sci Data. 2024 Mar 2;11:265. doi: 10.1038/s41597-024-03083-9 (PMC10908799; doi:10.1038/s41597-024-03083-9)
Supplement: Supplementary file 1 — Supplementary file [file 41597_2024_3083_MOESM1_ESM.pdf]

Supplementary Information

Contents

A Manual Evaluation and IAA Results in GARE Extraction **2**

B Analysis of Workflow Impact Upon GARE Extraction **3**

B.1 Sentence splitting . . . 3

B.2 Cascade errors affecting precision and recall rate in RE and GARE extraction tasks . . . 3

C Comparison of the Proposed Pipeline and LLM Method **5**

C.1 Prompt engineering by using GPT model . . . 5

C.2 Result comparison . . . 6

D Guideline of Code Usage **7**

D.1 Environment configuration . . . 7

D.2 External Tools . . . 7

D.3 Pipeline usage . . . 7

A Manual Evaluation and IAA Results in GARE Extraction

We recruit four human experts from the fields of biology, bioinformatics, and biomedical natural language processing to evaluate the quality of the GARE events in Cancer-Alterome. First, we utilize a small size set to evaluate the inter-annotator agreements among annotators and then set up a medium size set for GARE extraction evaluation. In the evaluation of the GARE extraction in the medium-size set, we mainly evaluate the efficacy of the template matching strategy, and the testing set is built upon sentences passing NER and NE. The details of the evaluation steps are in the following.

- IAA on GARE evaluation among four annotators.
  We randomly select 101 sentences that include GARE. Each annotator is required to extract the GARE result from the sentences. We then compare the GARE results among four annotators and compute IAA value.

- Calculation of the true positive (TP) and false positive (FP) in GARE extraction.
  We randomly select 1000 sentences that pass all the automatic processes of NER, RE, and GARE extraction. Annotators evaluate each sentence, observing the structure of GARE, including gene, genetic alteration, and the downstream effects. Annotators label a GARE with TP if the structure correctly matches the template and maps to the regulatory event(s); FP otherwise.

- Calculation of the true negative (TN) and false negative (FN) in GARE extraction.
  We randomly select 1000 sentences that pass the NER and RE process, but do not pass the GARE extraction. Annotators evaluate each sentence, observing whether there is a complete GARE in the sentence. Annotators label a sentence with FN if it includes a GARE; TN otherwise.

The IAA results are listed in Table S1, where Table S1 (a) provides IAA results with consistency percentage between annotators, Table S1 (b) provides Cohen Kappa metric, and Table S1 (c) provides the Fless Kappa metric. With all three metrics, the manual check-in GARE extraction achieves sufficient agreement among annotators. Here, Annotator 1 is the principal annotator, and the rest three are fellow annotators. Comparatively, higher consistency is obtained from the principal annotator and the annotators 2 and 4.

(a) IAA with consistency percentage

	Annotator 1	Annotator 2	Annotator 3
Annotator 2	0.9406		
Annotator 3	0.9010	0.9208	
Annotator 4	0.9109	0.9307	0.8911

(b) IAA with Cohen Kappa

	Annotator 1	Annotator 2	Annotator 3
Annotator 2	0.8811		
Annotator 3	0.7819	0.8418	
Annotator 4	0.8219	0.8614	0.8039

(c) IAA with Fless Kappa between 4 annotators

	Four annotators
Fless Kappa	0.8321

Table S1. IAA in manual check of the GARE extraction

By recruiting the annotators to manually evaluate 2000 GARE outcomes by the template-matching strategy, 842 out of 2000 GARE are tagged as TP, and 965 of them are tagged as TN. The complete confusion matrix result is given in Table S2, leading to a precision value of 0.84, a recall value of 0.96, and an F1 score of 0.90 in Table 2 (c).

		True GARE	
		Positive	Negative
Template matching	Positive	TP=842	FP=158
	Negative	FN=35	TN=965

Table S2. Confusion matrix for GARE extraction

B Analysis of Workflow Impact Upon GARE Extraction

B.1 Sentence splitting

For the selected 2000 sample sentences, we manually check the sentence splitting result by applying the pipeline. In total, 1943 out of them have correct sentence splitting results, and 57 of them have mistakes in the splitting. The wrong split mainly comes from the period used for abbreviations. For example,

"*However, a slight increase in pESR1ser167 was noted in the LTED model (Fig. 3b).*" (pmid: 29192207)

"*Stress exposure is related to increased expression of pro-inflammatory genes and reduced expression of interferon response genes that are important for coordinating the immune response to novel pathogens (Cole, Hawkley, Arevalo, & Cacioppo, 2011; Slavich & Cole, 2013; i.e., genes that regulate against viral infections).* " (pmid: 29548994)

or

"*Next, we analyzed the REV3L promoter sequence and identified a putative p73 binding site (BS) (Suppl. Figure S2).*" (pmid: 35519003)

We evaluate the impact of sentence splitting on GARE extraction. The contingency table is given in Table S3. Fisher's exact test provides a p-value of 1.00, which indicates that the sentence split result does not significantly affect the GARE extraction. Furthermore, an odds ratio of 1.0140 is derived, which indicates that the performance of GARE extraction is equally likely to be derived in a sentence with a correct split and one with an incorrect split.

	Correctly split sentences	Wrongly split sentences	Row total
With correct GARE	1741	51	1792
With incorrect GARE	202	6	208
Column total	1943	57	2000

Table S3. Contingency table in the Fisher's exact test.

B.2 Cascade errors affecting precision and recall rate in RE and GARE extraction tasks

Though the pipeline achieves competitive performance in NER, RE, and GARE extraction tasks, the overall evaluation is of importance for future improvement. Since the GARE extraction pipeline is based on a workflow strategy, cascade errors may affect the final performance. Therefore, we focus on 158 false positive cases and 35 false negative cases out of 2000 cases and analyze the main error impacts raised in NER or RE step. Generally, the 158 FP cases are cases that wrongly pass the NER, RE, and GARE extraction in the pipeline, whereas the 35 FN cases are cases that pass the NER and RE steps in the pipeline but are wrongly rejected in the GARE extraction step. The main reasons for the cascade errors are categorized into the following.

1) Missing NER taggings may bring silence in RE, and GARE extraction.

- A missing NER result will not affect GARE extraction if the sentence does not include a GARE. For example, in the sentence "*CDK2 is reported to induce phosphorylation of Skp2 on Ser 64 in the G1 phase.*"(pmid: 32014608), the gene tagging "CDK2" is missing. However, since there is no mention of mutation on CDK2 and this sentence does not match the GARE template, the absence of "CDK2" annotation will not affect GARE extraction.
- The missing NER tagging will affect GARE extraction if the entity is a part of an existing GARE. For example, in the sentence "*Alterations in the expression and signaling pathways of vascular endothelial growth factor have been linked to the clinical features and pathogenesis of hematologic malignancies.*"(pmid: 18987662), there exists a GARE, "*Vascular endothelial growth factor-Alterations-REGULATION-hematologic malignancies*". When the tagging of the entity "vascular endothelial growth factor" is missing, the extraction of the GARE fails.

2) Incorrect NER taggings bring the noise in RE, and may bring the noise to GARE.

- The error will not affect GARE extraction if the sentence does not contain a GARE. For example, in the sentence "*Functional studies have shown that the expression of CD200 on AML blasts could promote Treg formation while suppress the function of NK and memory T-cells.*" (pmid: 32002295), "memory" is wrongly tagged. Instead, the full tagging should be "function of NK and memory T-cells". However, since there does not exist a mutation, the sentence does not match the template. Therefore, the wrong tagging result does not lead to a false positive outcome.
- The error will affect GARE extraction if the sentence contains a GARE. For example, in the sentence "*For instance, epidermal growth factor receptor (EGFR) mutation can lead to the resistance of non-small cell lung cancer to Gefitinib by preventing this EGFR inhibitor from binding to its target.*" (pmid: 28981626), there is a GARE: "*EGFR – mutation – REGULATION – the resistance of non-small cell lung cancer*". However, the NER tagging merely tagged half part of the downstream biological process, leading to noise in GARE extraction.

3) Correct NER and incorrect RE results may bring noise to GARE extraction.

- The RE error will not affect GARE extraction if the sentence does not contain a GARE. For example, in the sentence "*The mutations resulted in an association between the phosphorylation of STAT3 and its localization in the nucleus.*" (pmid: 24709009), the relationship between "mutation" and "STAT3" was incorrectly identified as the *Theme*, resulting in an incorrect GARE. However, since this sentence does not contain information about the gene corresponding to "mutation", it does not match the template. Therefore, the incorrect RE result will not lead to false positives.
- The RE error will affect GARE extraction if the sentence contains a GARE. For example, in the sentence "*RNAi-mediated ablation of USP3 leads to the accumulation of DNA breakage and thus replication stress by delaying progression of the S-phase during cell division.*" (pmid: 34814083), there is a GARE: "*USP3 – ablation – REGULATION – accumulation of DNA breakage*". However, RE incorrectly marked the *Cause* relation between "ablation" and "cell division", introducing noise in GARE extraction.

4) Wrong trigger word recognition also brings noise in GARE.

- Negation are main cause for the wrong recognition of trigger words. For example, in the sentence *"All the ten investigated METTL3 SNPs rs3752411, rs1263793, rs2242526, rs1268403, rs113058369, rs1263797, rs1263796, rs11851342, rs1263800 and rs10450908 <u>were not associated</u> with colorectal cancer risk. "* (pmid: 32615646), it mentions that mutations *occurs in* METTL3 "were not associated with" colorectal cancer risk. However, a missing tagging of the underlined negation leads to an incorrect GARE, e.g., "*METTL3 – rs3752411 – REGULATION – colorectal cancer risk*".

C Comparison of the Proposed Pipeline and LLM Method

C.1 Prompt engineering by using GPT model

In the LLM evaluation, we formulated task-specific prompts to query the gpt-3.5-turbo model through the OpenAI API. The resulting responses are subsequently evaluated.

As illustrated in **Figure S1**, the designed prompts typically consist of five components. The task definition section is employed to assist ChatGPT in understanding the definition of the task. The extraction rules section outlines specific requirements for the task, such as entity boundaries in NER task. The format requirements section specifies the desired format for the returned response data, facilitating subsequent processing. The examples part provides several annotated cases to further aid the LLM in comprehending the task objectives. The empty sentences section, on the other hand, supplies blank sentences to be annotated in bulk. All prompts and scripts can be found in the GitHub repository.

Taking the GARE extraction task as an example, the prompt is designed as below.

- **Task Definition:** *We define "genetic alteration caused regulatory event" as defined below. A genetic alteration occurs in a specific gene, then the alteration further causes the regulation of a downstream biological process. There are three different regulatory types, neutral regulation (Reg), positive regulation (PosReg), and negative regulation (NegReg). Finally, event are formed as "gene – genetic alteration – trigger word (regulatory type) – biological process"*

- **Extraction Rules:** *Rules for extraction: 1. The gene in the event should be an isolated gene name or symbol, not containing other words, like "gene", or "mutated". 2. One event should contain only a single gene and a single biological process. 3. The genetic alteration in an event should be the change of the gene, like SNPs, or general alteration, e.g. "mutant", or "knockdown". 4. The trigger words in the event should be divided into three regulatory types, e.g. "cause" is neutral regulation, "enhanced" is positive regulation, and "reduced" is negative regulation. 5. The biological process in the event should be an isolated concept, not containing other adjectives, like "enhanced" and "different", and it's not always the predicate in the sentence. 6. Trigger words keep only the most important words. 7. The event has to include 4 elements, gene, genetic alteration, trigger word, and biological process. 8. All elements in the event must be in the sentence. 9. If the event describes negation, mark "Negation" in the regulatory type, e.g. "no association".*

- **Format Require:** *Please respond to me in the following format:*

sent-1:

event-1: gene1 – genetic alteration1 – trigger word1 (regulatory type1) – biological process1

- **Examples:** *Some example:*

sent-1: 23752191 Downregulation of miR-140 promotes cancer stem cell formation in basal-like early-stage breast cancer.

event-1: miR-140 – Downregulation – promotes (PosReg) – cancer stem cell f ormation

- **Empty Sentences:** *Please extract the events from the following sentences: <target sentences>*

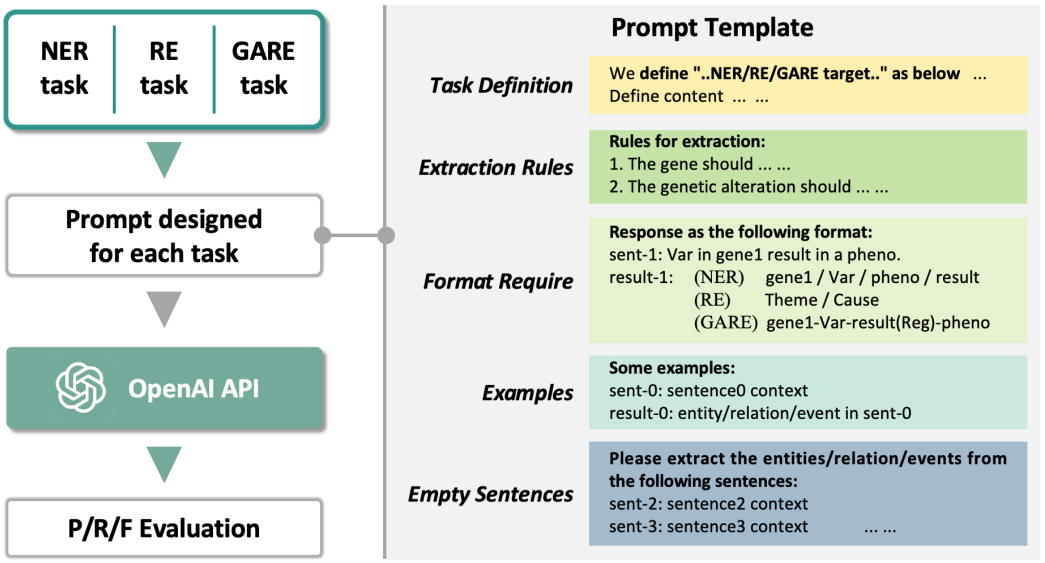

Figure S1. Evaluation strategies of OpenAI LLM used to pipeline task.

C.2 Result comparison

As shown in **Table S4**, the experimental results indicate that LLM is generally inferior to the existing tools and methodology used in our pipeline in almost all scenarios. The only exception is in NER for GO, where LLM outperforms OGER++ by 0.01 in terms of F1-score. However, considering the disadvantage of LLM in precision, it is still wise to use OGER++ in the pipeline to ensure the reliability of the results.

(a)

**Evaluation metric**		**Named entity recognition and normalization**		
Entity type	Tools	Precision	Recall	F1 Score
Gene	PubTator[20] v.s. ChatGPT method[36]	**0.79** v.s. 0.32	**0.81** v.s. 0.46	**0.80** v.s. 0.38
Point mutations and SNPs	PubTator[20] v.s. ChatGPT method▲	**0.81** v.s. 0.72	**0.81** v.s. 0.68	**0.83** v.s. 0.70
General genetic alteration	AGAC-NER[24] v.s. ChatGPT method▲	**0.74** v.s. 0.08	0.57 v.s. **0.72**	**0.64** v.s. 0.14
Trigger word	AGAC-NER[24] v.s. ChatGPT method▲	**0.78** v.s. 0.16	**0.70** v.s. 0.40	**0.74** v.s. 0.23
GO	OGER++[22] v.s. ChatGPT method▲	**0.72** v.s. 0.29	0.17 v.s. **0.26**	0.27 v.s. **0.28**
HPO	PhenoTagger[23] v.s. ChatGPT method▲	**0.79** v.s. 0.22	**0.70** v.s. 0.22	**0.74** v.s. 0.22
MeSH	PubTator[20] v.s. ChatGPT method[37]	**0.83** v.s. 0.50	**0.82** v.s. 0.51	**0.81** v.s. 0.50

(b)

**Evaluation metric**		**Relation extraction**		
Relation type	Tools	Precision	Recall	F1 Score
Theme	AGAC-RE[24] v.s. ChatGPT method▲	**0.87** v.s. 0.35	**0.84** v.s. 0.53	**0.91** v.s. 0.25
Cause	AGAC-RE[24] v.s. ChatGPT method▲	**0.88** v.s. 0.33	0.85 v.s. **0.91**	**0.82** v.s. 0.28

(c)

**Evaluation metric**		**Regulatory events identification**		
Event type	Method	Precision	Recall	F1 Score
GARE	Template match v.s. ChatGPT method▲	**0.84** v.s. 0.65	**0.96** v.s. 0.51	**0.90** v.s. 0.57

▲: ChatGPT-based method with our prompt design.

Table S4. Performance comparison between the proposed pipeline and ChatGPT-based method. (a) NER evaluation. (b) RE evaluation. (c) GARE evaluation.

In all remaining scenarios where LLM prevails, its superiority is restrictively in the Recall metric for GO-NER and *Causs*-RE. These findings suggest that, in the discipline of specialized academic vocabulary and terminology, LLM is not fully capable of competing with conventional methods in terms of Precision. However, there is a potential to leverage its extensive pre-training data to enhance Recall for certain text-mining tasks. Considering the critical reliance on accuracy in the context of biomedical knowledge extraction, we prefer to adopt the proposed pipeline and afford greater result reliability.

D Guideline of Code Usage

The complete pipeline implementation as well as the data visualization script can be found in the GitHub repository, `https://github.com/bionlp-hzau/Cancer-Alterome`.

D.1 Environment configuration

The pipeline implementation is based on Python 3.6, and all code is provided as Python scripts or Jupyter scripts.

The code relies on a number of common Python libraries, e.g. Numpy, Pandas, NLTK and PyEcharts. All libraries can be installed via *pip install <library>* or *conda install <library>*.

D.2 External Tools

The pipeline implementation involves several external tools, the function description and quick link of these tools are provided below. Users may need to configure and test these tools before running the full Cancer-Alterome pipeline.

- PubTator Central: This tool is used to identify and standardize genes, point mutations, single nucleotide mutations, and disease mentions in the pipeline.
- AGAC-NER: This model is used to identify the genetic alteration and trigger word defined in AGAC corpus.
- AGAC-RE: This model is used to extract the relations defined in AGAC corpus, include *Theme* and *Cause*.
- OGER++: This tool is used to identify the mentions of GO concepts in the text, and normalize mentions to GO IDs.
- PhenoTagger: This tool is used to identify the mentions of HPO concepts in the text, and normalize mentions to HPO IDs.

D.3 Pipeline usage

All 16 backbone scripts for each step in the pipeline are released in the GitHub repository, and the script names are numbered indicating the running order. The usages of the script are listed below.

D.3.1 Scripts for literature preparation

Four scripts are involved in the literature preparation step.

- *1. esearch_get_pmc_pmid.py* is used to query PubMed and PubMed Central databases based on the keywords, and to download PMID and PMCID automatically.
- *2. pmc_pmid_to_biocjson.py* is used to download the abstracts as well as the full text of the corresponding literature and PubTator annotations from the PubTator API based on the PMIDs and PMCIDs.
- *3. biocjson_to_pubtator.py* provides PubTator format conversion of BiocJson format files. This conversion step is necessary due to the pipeline design where the PubTator format is used for subsequent data processing.
- *4. biocjson_to_jounral_info.py* is used to extract the journal information of the article from the BiocJson format file, e.g. journal name, the year of publication.

D.3.2 Scripts for named entity recognition and normalization

Six scripts are included in this step, and the usages are as below.

It should be noted that since AGAC-NER already has isolated repositories, only the format conversion scripts for its input and output are provided in Cancer-Alterome repositories.

In addition, before using OGER++ and PhenoTagger, the environment of these two tools should be well configured.

- *1. pubtator_to_agac_ner_input.py* provides the conversion of the PubTator format file to the input format of the AGAC-NER task.
- *2. agac_ner_output_procss.py* is used to process the output file of the AGAC-NER task.
- *3. oger_tagger.py* provides batch GO concept annotation by using OGER++. The vocabulary files required for OGER++ are also provided in go.term.tsv and hpo.term.tsv.
- *4. OGER_result_process.py* provides the result processing for OGER++ results.
- *5. PhenoTagger_training.py* provides the model training function of PhenoTagger.
- *6. PhenoTagger_tagging.py* is used to batch tag the HPO concepts by using PhenoTagger.

D.3.3 Scripts for relation extraction

Two scripts are included in this step to perform relation extraction tasks based on previous results.

Similarly, the AGAC-RE model should be well configured before running the following script.

- *1. ner_tagging_to_agac_re_input.py* provides the format conversion of the AGAC-NER output to the AGAC-RE inputs.
- *2. agac_re_infer_process.py* is used to process the output files of the AGAC-RE task.

D.3.4 Scripts for regulatory events extraction

One script is used in this step to perform the rule-based GARE extraction.

- *1. rule_based_gare_extraction.py* is used to extract the complete GARE by using designed rules.

D.3.5 Scripts for data visualization

Three scripts included in this step for generating the data visualization shown in the paper.

- *1. gare_to_database.py* is used to convert the GARE results generated in the previous step into a more readable tab-delimited database format.
- *2. multi-gene-heatmap.ipynb* is used to plot the heatmap web visualization shown in the paper.
- *3. Single_gene_SanKey.ipynb* is used to plot the SanKey web visualization presented in the literature.

D.3.6 Scripts and prompt for LLM evaluation

There are three scripts and five prompt files are used for LLM performance evaluation.

- *1. gpt_query_xzyao.py* used to batch request the OpenAI API using prompt and save the response results.
- *2. chatgpt_ner_eval.py* used to evaluate the NER annotation results returned by the API.
- *3. chatgpt_re_eval.py* used to evaluate the RE annotation results returned by the API.
- *4. event-generation-evaluation.py* used to evaluate GARE generation results returned by the API.
